# Membrane damage mechanism of protocatechualdehyde against *Micrococcus luteus* and its effect on pork quality characteristics

**DOI:** 10.1038/s41598-022-23309-3

**Published:** 2022-11-07

**Authors:** Sichen Liao, Guoli Gong, Xuyang Wang, Lu Tian

**Affiliations:** School of Food and Biological Engineering, Shaanxi University of Science and Technology, Xi’an, 710021 Shaanxi China

**Keywords:** Antimicrobials, Applied microbiology, Infectious-disease epidemiology

## Abstract

This study investigated the mechanism of membrane damage by protocatechualdehyde (PCA) against *Micrococcus luteus* and assessed effects of PCA on the sensory and physicochemical properties of pork. The mechanism of PCA inhibition on *M. luteus* was studied by determining the minimum inhibitory concentration (MIC) based on membrane potential, intracellular ATP concentration, intracellular pH, confocal laser scanning microscopy (CLSM), and field emission gun scanning electron microscopy (FEG-SEM). The results showed that the MIC of PCA against *M. luteus* was 1.25 mg/mL. Hyperpolarization of the bacterial cell membrane, a decrease in the intracellular ATP concentration, and intracellular pH indicated that PCA damaged the cell membrane of *M. luteus*. FEG-SEM observation revealed that PCA could cause surface collapse, cell membrane rupture, and content outflow of *M. luteus*. Additionally, PCA was found to inhibit increases in the total number of colonies, the thiobarbituric acid reactive substances (TBARS) value growth rate, and moisture mobility in raw pork. Additionally, it improved the color and texture of raw pork, all of which effectively prolonged its shelf life. This study will encourage the application of PCA as a natural antibacterial agent in the food industry.

## Introduction

During the storage process, food products can be easily contaminated by microorganisms, which can cause spoilage and deterioration, and preservatives have typically been added to extend the storage period of food. However, chemical preservatives have potentially toxic side effects and residual problems, and can pose a hazard to human health if they are improperly added. Additionally, excess use can cause environmental pollution^1^. As such, the development of green, safe, and efficient natural food preservatives is a popular research topic. Natural plant-derived preservatives are rich in resources, environment-friendly, healthy, safe, and non-toxic, and have antibacterial properties^2^. They can serve as new preservatives and antibacterial agents, which can be used in flavor and fragrance, cosmetics, food, medicine, and pharmaceuticals^3^. Plant-derived antimicrobial agents have been reported, including polyphenolic compounds (e.g., green tea polyphenols, oleuropein, vanillic acid, cinnamic acid, ferulic acid, and syringic acid), flavonoids (e.g., quercetin), and terpenoids (e.g., carvacrol, thymol, eugenol)^4^. They have high antibacterial properties and can effectively preserve food. Using the antioxidant and antibacterial properties of olive leaf essential oil, Saleh et al.^5^ found that adding olive leaf extract (OLE) to poultry meat could inhibit the growth of microorganisms, maintain the chemical quality and sensory properties of poultry meat, and extend the shelf life of poultry meat. Tamkutė et al.^6^ found that effectively isolated cranberry pomace extracts inhibited the growth of food-borne pathogens and the formation of lipid oxidation products (malondialdehyde) in pork, which increased the microbiological safety and oxidative stability of pork products. In recent years, protocatechualdehyde (PCA) has attracted attention for its multiple effects. In the field of food preservation, PCA has scientific value and various prospective applications as a natural food additive to extend the shelf life of certain products.

PCA, a natural water-soluble antioxidant compound, belongs to the class of phenolic acids. PCA is identified in a variety of herbs (e.g., gall leaves, holly leaves and salvia roots), most of which are wild^7^. Chang et al. identified six major compounds from the ethyl acetate extract of *Phellinus linteus*. They concluded that PCA is the major phenolic compound of *P. linteus* with DPPH radical scavenging activity, among other effects^8^. Preliminary studies have suggested that PCA exhibits various biological activities (e.g., anti-atherosclerosis, anti-oxidation, anti-inflammatory, anti-fibrosis, anti-cancer, anti-tumor, cardiovascular protection, nerve protection, and other effects)^9–11^. Existing studies have suggested that PCA exhibits various antibacterial properties. PCA displays suitable antibacterial activity against food-borne pathogens such as *Staphylococcus aureus*, *Bacillus cereus*, *Escherichia coli*, *Ralstonia solanacearum*, and *Cronobacter sakazakii*^12–14^. However, the inhibitory effect of PCA on *Micrococcus luteus* and its mechanism and application as an antimicrobial agent added to food have been rarely reported.

*Micrococcus luteus*, a Gram-positive coccus of the genus Micrococcaceae, generally divides singly, in pairs, and multiple directions to form tetrads or irregular three-dimensional colonies^15^. *M. luteus* is bacteria that can spoil food and has been detected in the air, water, soil, plants, and food. The bacterium can contaminate meat, fish, aquatic goods, soybean products, and other meals and has been identified in high concentrations in fresh, processed, and spoiled foods^16,17^. Humans infected with this bacteria can experience tissue inflammation, sepsis, shock, and other health hazards^18,19^.

This study aimed to reveal the membrane damage mechanism of PCA on *M. luteus*, and to evaluate the effects of PCA on the colony number, color, TBARS value, TPA, moisture distribution, and sensory properties of raw pork containing *M. luteus* on the 1st, 3rd, 5th, and 7th day after exposure. This study provides a theoretical basis for developing PCA as a natural antibacterial agent and food additive.

## Materials and methods

### Reagents

PCA [HPLC ≥ 98%, CAS: 139-85-5] was provided by Bioengineering Co., Ltd (Shanghai). After preparing the PCA stock solution with ethanol as a solvent, the solution was dissolved in Mueller–Hinton II Broth (CA-MHII), Luria–Bertani medium (LB), and Phosphate adjusted Buffered Saline (PBS). The final reaction system contained 2% ethanol. It was then filtered by a 0.22 μm filter and stored at − 20 °C. All additional chemicals used to make the buffers (acid-hydrolyzed casein, beef powder, soluble starch, anhydrous calcium chloride, Na_2_HPO_4_·12H_2_O, KH_2_PO_4_, K_2_HPO_4_, NaCl, KCl, and MgCl_2_) were of analytical grade.

### Bacterial strain and culture conditions

The *Micrococcus luteus* BNCC 102589 strain originated from BeNa Culture Collection (BNCC, Beijing, China) and was stored in glycerol bottles at − 80 °C. They were then inoculated into Luria–Bertani (LB) medium for activation and cultured at 37 °C for 18 h. The bacteria grew to the logarithmic growth phase (approximately 10^8^ CFU/mL). All experiments were performed with final cultures of *M. luteus*.

### Raw meat pretreatment

The fresh pork ham was minced twice with a muscle mincer equipped with 6 mm and 3.2 mm orifice plates. Therefore, the minced pork was fully mixed and placed under a high-power Ultraviolet Radiation A-Light Emitting Diode (UVA-LED) (NCCU033(T); Nichia Corporation, Japan, wavelength 365 nm) for 30 min for sterilization. Bacterial cells were collected by centrifugation at 8000×*g* for 5 min, and PCA solutions of 0, MIC, and 2 × MIC were added to the precipitate containing bacterial cells (PCA solutions were prepared with PBS supplemented with 2% ethanol). PBS supplemented with 2% ethanol was used as a negative control. The final concentration of ampicillin for a positive control was 0.1 mg/mL (ampicillin was prepared with sterile water). Subsequently, 1 mL of each solution was aspirated and injected into 100 g of ground pork. It was then mixed well, covered in PVC film packing, and refrigerated at 4 °C. The total number of colonies and changes in quality were examined on the 1st, 3rd, 5th, and 7th days after exposure.

### Minimum inhibitory concentration determinations

MIC was determined using a modified broth microdilution method by Tian et al.^20^. CA-MHII solution supplemented with 2% ethanol was prepared with different concentrations of PCA solution (5, 2.5, 1.25, 0.625, 0.3125, 0.15625, 0.078, 0.039, 0.0195, 0 mg/mL). CA-MHII solution supplemented with 2% ethanol was used as a negative control. The final concentration of ampicillin for the positive control was 0.1 mg/mL. A total of 100 μL of bacterial culture and 100 μL of PCA solution were added to 96-well plates (Nunc, Copenhagen, Denmark). The plates were then incubated for 8 h by adjusting the concentration of the PCA solution. The MIC of PCA on *M. luteus* was determined by examining the absorbance value at 600 nm with a full wavelength scanning multifunctional reader (Varioskan Flash, Thermo Fisher, Finland).

### Growth curves

The growth curve of *M. luteus* was determined according to the method described by Shi et al.^14^. PCA solutions from 2 × MIC to 1/128 × MIC were prepared with LB broth supplemented with 2% ethanol. LB broth supplemented with 2% ethanol was used as a negative control. A total of 100 μL of bacterial culture and 100 μL were added to a 96-well plate (Nunc, Copenhagen, Denmark). The absorbance was examined every 1 h to plot the growth curve of the cells for 24 h.

### Membrane integrity and membrane potential

In accordance with the modified protocols used by Guo et al.^21^, the bacteria cells were washed 2–3 times with PBS solution, and the cells were resuspended by centrifugation in PCA solutions at concentrations of 0, MIC, and 2 × MIC. PCA solutions were prepared from LB broth supplemented with 2% ethanol. LB broth supplemented with 2% ethanol was used as a negative control. Subsequently, 200 μL of the cell suspension was added to a black opaque 96-well microtiter plate (Nunc, Copenhagen, Denmark) and incubated for 2 h. Finally, 2 μL of 1 mM of the fluorescent probe bis-(1,3-dibutylbarbituric acid) trimethineoxonol [DiBAC4(3); Molecular Probes, Sigma, Louis, USA] was added and incubated for 10 min. The fluorescence intensity was examined at 492 nm excitation/515 nm emission wavelength with a multi-mode reader (Synergy H1, BioTek, Vermont, USA).

### Determination of intracellular ATP

The intracellular ATP was detected by an ATP assay kit (Beyotime Bioengineering Institute, Shanghai, China) according to the method used by Bajpai et al.^22^.

### Intracellular pH measurements

Based on the method of Shi et al.^23^, the bacterial pellet was washed twice with HEPES buffer, and the CFDA-SE fluorescent probe (final concentration was 3 μmol/L) was added to the bacterial suspension and incubated for 20 min. Subsequently, the cells were washed once with potassium phosphate buffer, resuspended, and incubated with glucose (final concentration was 10 μmol/L) for 30 min. Lastly, the bacteria pellets were washed twice with potassium phosphate buffer solution, and the fluorescence-labeled cells were resuspended with PCA solution prepared with PBS supplemented with 2% ethanol (concentrations of 0, MIC, and 2 × MIC) and cultured in the dark for 1 h. PBS supplemented with 2% ethanol was used as a negative control.

The samples and the standard curve set were added to a black enzyme standard plate, and the fluorescence intensity of the samples was detected at 490 nm excitation/520 nm emission wavelength and 440 nm excitation/520 nm emission wavelength. The intracellular pH was obtained in accordance with the standard curve.

### Confocal laser scanning microscopy (CLSM) examinations

Cell membrane permeability was examined with a LIVE/DEAD BacLight™ Bacterial Viability Kit (Molecular Probes, Thermo Fisher, USA) according to methods used by Tian et al.^20^.

### Field emission gun scanning electron microscopy (FEG-SEM) analysis

Cell morphology was observed using the method described by Su et al.^24^. Bacterial suspensions were treated with PCA solutions (concentrations of 0, MIC, and 2 × MIC) and prepared with PBS supplemented with 2% ethanol for 24 h at 37 °C. PBS supplemented with 2% ethanol was used as a negative control. Next, cells were washed 2–3 times with PBS and fixed in 2.5% glutaraldehyde-PBS solution. After 12 h, they were washed 2–3 times. Afterward, they were dehydrated in ethanol solutions with different concentration gradients (30–100%) for 10 min, suspended in isoamyl acetate for 30 min, and dried. Lastly, the dried samples were fixed on silicone film, surface gold sprayed, and photographed using high-resolution field emission scanning electron microscopy (MLA 650, FEI, Hillsboro, USA).

### Simulation study of PCA on the growth inhibition of *M. luteus* in pork

The samples were treated as described in “Raw meat pretreatment” section using the method of Zhang et al.^25^, with modifications. The Box–Behnken design test was performed with PCA concentration (A), storage temperature (B), and storage time (C) as the variables. The treated samples were diluted and spread on LB agarin (Table 1), and the actual number of colonies was recorded. The response surface methodology was adopted to build and analyze the growth inhibition model of PCA on *M. luteus* in pork based on different parameters.

**Table 1:** Box–Behnken experimental design and responses.

Trials	*A* (day)	*B* (℃)	*C* (mg/mL)	log_10_ (N/(CFU/g))
1	1	4	1.25	5.48
2	5	4	1.25	5.67
3	1	37	1.25	5.56
4	5	37	1.25	5.72
5	1	20.5	0	5.69
6	5	20.5	0	5.98
7	1	20.5	2.5	5.43
8	5	20.5	2.5	5.45
9	3	4	0	5.66
10	3	37	0	5.91
11	3	4	2.5	5.42
12	3	37	2.5	5.41
13	3	20.5	1.25	5.73
14	3	20.5	1.25	5.75
15	3	20.5	1.25	5.79
16	3	20.5	1.25	5.86
17	3	20.5	1.25	5.89

### Color measurement

The method described by Fan et al.^26^ was adopted. Samples of minced pork with a thickness of 1 cm were obtained. A CR-600 colorimeter (Minolta, Osaka, Japan) was used for zero point correction and whiteboard correction, and changes in the luminance (L*), redness (a*), and yellowness (b*) values of each group were observed on the 1st, 3rd, 5th, and 7th days, respectively.

### Determination of texture profile analysis (TPA)

The ground pork was shaped into a figure nearly 1 cm in height and approximately 2 cm in diameter at the bottom, in accordance with the improved protocols used by Novakovi et al.^27^. The TA Plus physical property tester (texture profile analysis, TPA) was used for testing. The P/75 probe model was selected at a compression ratio of 35%, a pre-test and test speed of 1 mm/s, and a post-test speed of 5 mm/s. A texture profile analysis (TPA) was conducted on the hardness, adhesiveness, gumminess, chewiness, springiness, cohesiveness, and resilience of the ground pork.

### Determination of thiobarbituric acid reactive substances (TBARS)

Using the method of Quevedo et al.^28^ with some modifications. 2.0–2.5 g of ground pork samples were accurately weighed and 1.5 mL of Thiobarbituric acid (TBA) solution (1% TBA dissolved in 0.75 mol/L sodium hydroxide) and 8.5 mL of Trichloroacetic acid (TCA) solution (2.5% TCA dissolved in 0.036 mol/L HCl) were added. After a water bath at 100 °C for 30 min and an ice water bath to cool it to an ambient temperature, 4 mL of supernatant was obtained and 4 mL of chloroform was added. The mixture was then shaken well. The absorbance of ABS was examined at 532 nm after centrifugation at 3000×*g* for 5 min. The results were expressed as malondialdehyde (MDA) equivalent (mg/kg), and the TBARS values were obtained as follows: TBARS (mg/kg) = *ABS/W* × 9.48.

### Low-field nuclear magnetic resonance (LF-NMR) T_2_ relaxation measurements

Using the method of Cai et al.^29^, the NMR relaxation test was performed at a proton resonance frequency of 21 MHz and a measuring temperature of 32 °C. Nearly 2 g of minced meat samples were placed in a 25 mm diameter NMR tube, which was then placed in the analyzer for measurement with the Q-CPMG sequence. The parameters applied include a sampling frequency of 100 kHz, 90° pulse width of 7 μs, 180° pulse width of 13.52 μs, waiting time of 1000 ms, analog gain of 20, a digital gain of 3, echo time of 0.4 ms, echo number of 12,000, and 8 repetitions of the scan. The *T*_2_ relaxation pattern was obtained by inverting the data with the inversion software.

### Sensory evaluation

Ground pork was formed into patties of approximately 1 cm in height and 2 cm in diameter at the bottom. The pork patty samples were then submerged in water for 10 min and boiled at high temperatures for 5 min. After being allowed to naturally cool to room temperature, a sensory panel of 10 students trained in sensory evaluation evaluated the color, texture, odor, taste and overall appreciation of pork patties for each storage period (1st, 3rd, 5th, and 7th day) on a 9-point happiness scale for sensory evaluation^30^. This method was used to determine the acceptability of pork patty samples treated with different concentrations of PCA and to verify the effects of PCA as a natural antimicrobial agent on the sensory properties of the food.

### Statistical analysis

All experiments were performed in three replicates, with each biological replicate including two technical replicates. The data analysis was performed with SPSS software (version 19.0; SPSS, Inc., Chicago, IL). A student’s t-test was used to verify differences between groups, which are expressed as mean standard deviation (n = 3). *P* ≤ 0.05 was considered statistically significant.

## Results and discussion

### MIC and growth curves of PCA to *M. luteus*

The MIC of PCA against *M. luteus* was 1.25 mg/mL. PCA as a natural extract was found with broad-spectrum antibacterial activity. For instance, the MIC of PCA against *Yersinia enterocolitica* was 0.3125 mg/mL^20^, the MIC against *C. sakazakii* was 1.25 mg/mL^14^, and the MIC against *S. aureus*, *B. cereus* and *E. coli* was measured as 0.9, 1.0 and 3.0 mg/mL, respectively^12^. The results of this experiment suggested that PCA also exhibited high antibacterial activity against *M. luteus*, which provides a theoretical basis for food contamination related to *M. luteus*.

**Figure 1:**
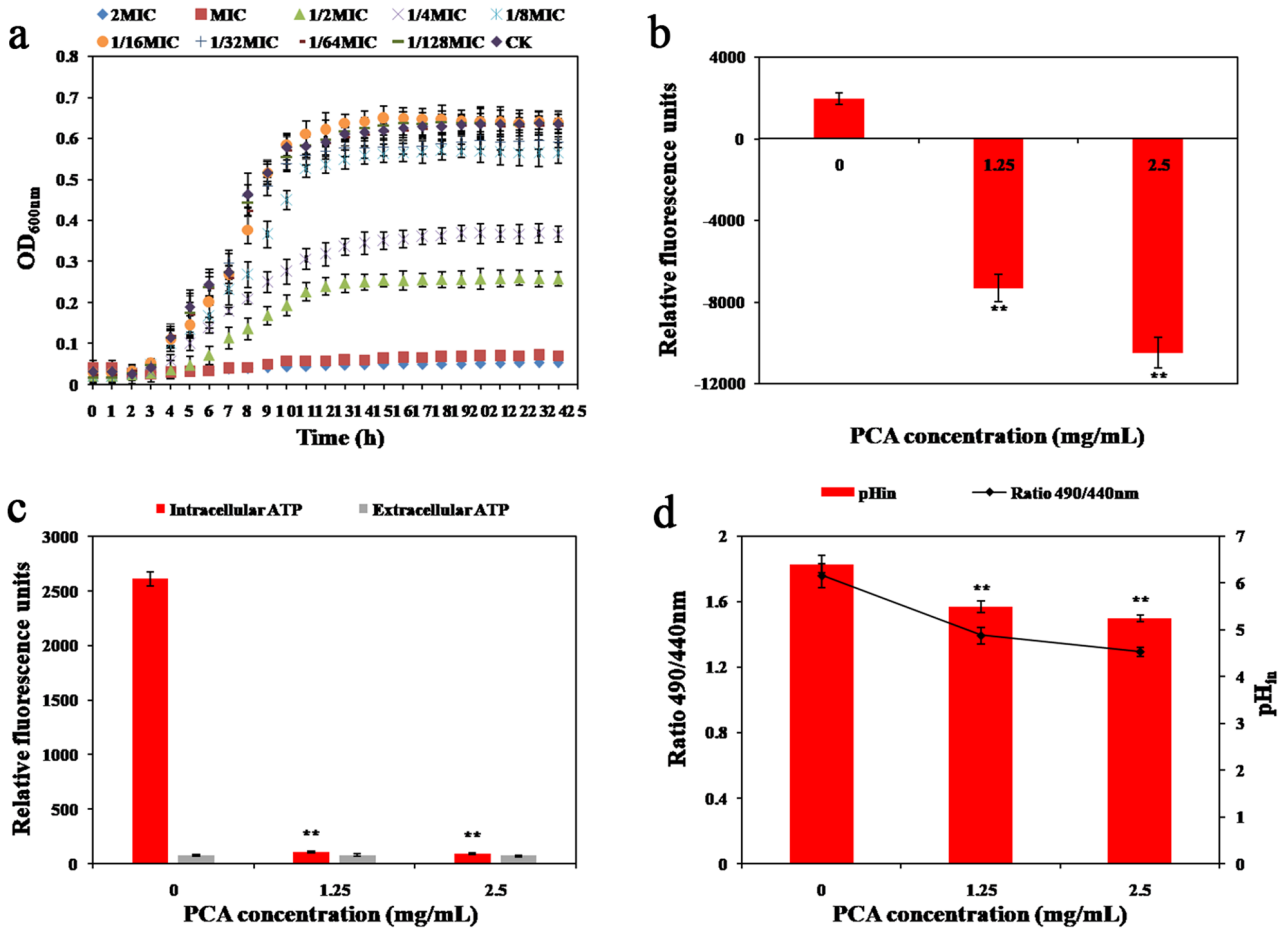
(**a**) The growth curves of *M. luteus* BNCC 102589 exposed to different concentrations of PCA. (**b**) The effects of PCA on the membrane potential of *M. luteus* BNCC 102589. (**c**) The effects of PCA on intracellular ATP levels of *M. luteus* BNCC 102589. (**d**) The effects of PCA on the pH_in_ of *M. luteus* BNCC 102589.

The inhibitory effects of PCA on *M. luteus* were explored through growth curve measurement, and the results were obtained based on the OD_600_ value. As depicted in Fig. 1a, the growth of *M. luteus* reached a stable phase after 10 h with an OD_600_ value of 0.63. PCA at 1/2 × MIC completely inhibited the growth of *M. luteus* within 24 h, and PCA at 1/4 × MIC and 1/8 × MIC partially inhibited its growth. Compared with the control group, the final bacterial concentration of *M. luteus* decreased by 2/3 under the treatment of PCA with 1/2 × MIC and by 1/2 under the treatment of PCA with 1/4 × MIC. Furthermore, the growth of *M. luteus* decreased as the PCA concentration increased. Therefore, low concentrations of PCA could more significantly inhibit the growth of *M. luteus*.

### Membrane potential

Membrane potential is the potential difference between the inside and outside of the cell membrane. Both depolarization and hyperpolarization of membrane potential could potentially damage the cell membrane. Accordingly, the maintenance of membrane potential serves as an indicator of membrane integrity^31^. Since changes in cell membrane potential can lead to changes in fluorescence light intensity, changes in membrane potential can be indirectly characterized by fluorescence intensity. DiBAC4(3) is a lipophilic anion that can penetrate cells with disordered membrane potential and bind to intracellular proteins or membranes^32^. As depicted in Fig. 1b, the fluorescence intensity of the cells significantly decreased (*P* < 0.05) when *M. luteus* was treated with PCA at MIC and 2 × MIC compared with the control group. This indicates that PCA can hyperpolarize the cell membrane of *M. luteus*. Similar studies showed that treatment of *Penicillium roqueforti* with eugenol combined with citra resulted in severe mitochondrial collapse and exhibited hyperpolarization of membrane potential^33^. Shi et al.^23^ treated *C. sakazakii* with ferulic acid and showed cell membrane hyperpolarization. The cell membrane hyperpolarization could be caused by K^+^ to maintain the equilibrium of the internal and extracellular potentials after the change of pH or cell membrane permeability^34^. Therefore, PCA could increase the permeability of bacterial cell membranes after acting on *M. luteus* and change the cell membrane potential, thus inhibiting *M. luteus*.

### Intracellular ATP concentrations

ATP plays a vital role in energy metabolism as a high-energy phosphate compound. Normal intracellular ATP levels are in a steady state. When the cell membrane is damaged, intracellular ATP metabolism becomes abnormal, and the ATP level in bacterial cells will rapidly decline. Thus, the ATP content is capable of indicating the survival status of the cell. In this experiment, firefly luciferase was used to evaluate the effects of PCA on the intracellular ATP concentration of *M. luteus*. As depicted in Fig. 1c, the intracellular ATP luminescence intensity of *M. luteus* treated with PCA at MIC and 2 × MIC was significantly lower (*P* < 0.05) compared with untreated cells, while the extracellular ATP luminescence intensity did not significantly change. This indicates that intracellular ATP was naturally depleted after PCA treatment and that the decrease in intracellular ATP concentration could be due to the disruption of cell morphology and increases in cell membrane permeability^21^. This is consistent with the findings of Shi et al.^14^, who treated *C. sakazakii* with 2 × MIC and 4 × MIC of PCA and found a decrease in intracellular ATP content in *C. sakazakii*. They reported that PCA caused an increase in the rate of ATP hydrolysis by *C. sakazakii* and could change the permeability of the cell membrane, thus inhibiting the bacteria.

### Intracellular pH measurements

Changes in pH_in_ can indicate bacterial cell damage. Cells with intact cell membranes can maintain a stable internal pH when there are moderate changes in external pH^35^. Figure 1d displays changes in the intracellular pH of *M. luteus*, as indicated by detecting fluorescence. The pH_in_ of normal *M. luteus* cells was 6.40 ± 0.18. The pH_in_ of *M. luteus* cells treated with PCA significantly decreased compared with the control group (*P* < 0.05). pH_in_ decreased from 6.40 ± 0.18 to 5.24 ± 0.07. These results align with those of Gonelimali et al.^36^, who found that treatment of *S. aureus* and *E. coli* with Ethanolic extracts of clove and water extracts resulted in a decrease in pHin. These changes indicate that the bacterial cell membrane was damaged. PCA affects the internal environment of *M. luteus* cells, leading to abnormal metabolic functions of these cells^35^.

### CLSM observation

**Figure 2:**
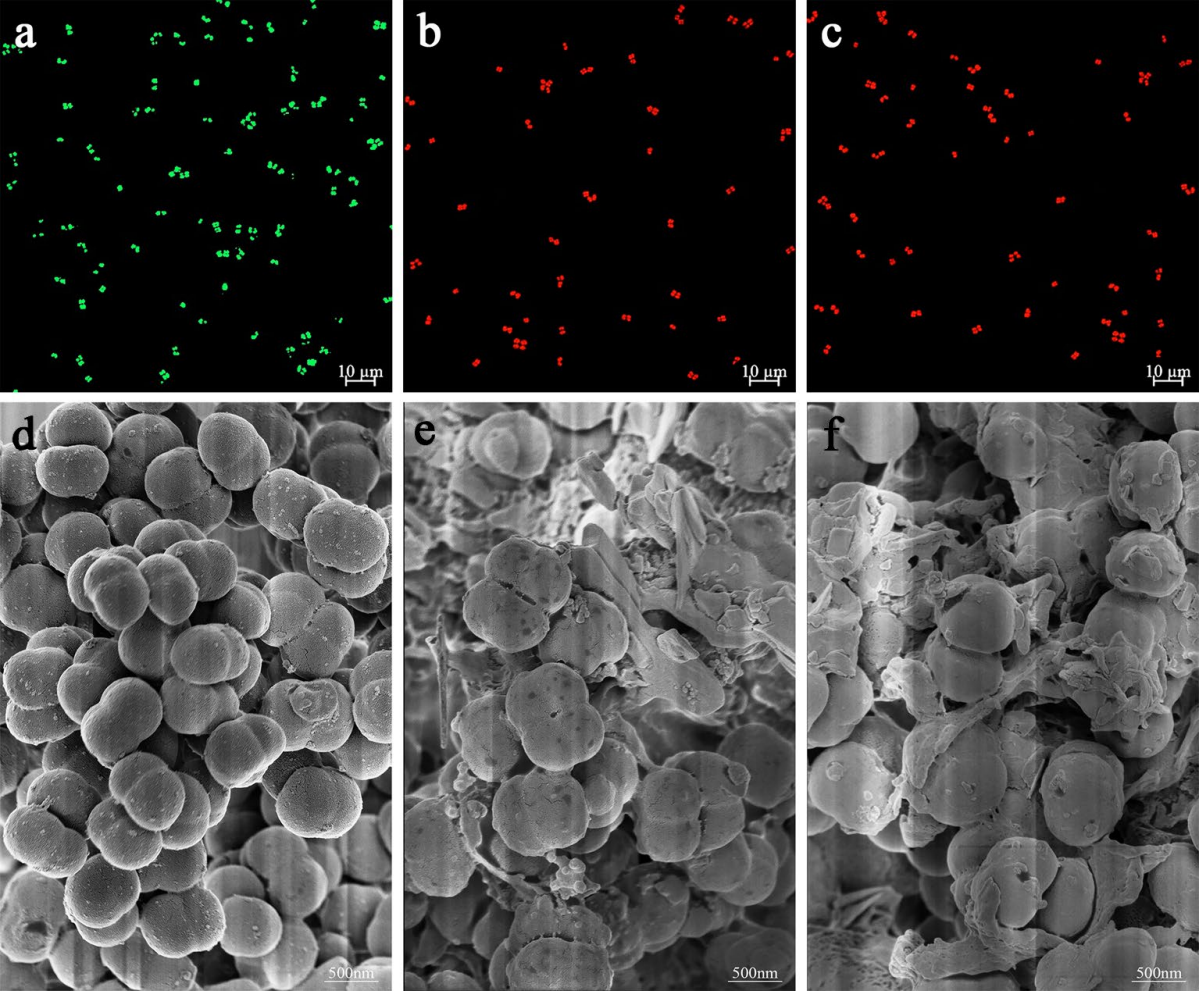
The effects of PCA on the cell membrane integrity of *M. luteus* BNCC 102589 by CLSM. *M. luteus* cells were treated with PCA at the (**a**) 0 × MIC, (**b**) MIC, and (**c**) 2 × MIC, respectively. The scanning electron micrographs of *M. luteus* BNCC 102589 under different treatments. *M. luteus* were treated with PCA at the (**d**) 0 × MIC, (**e**) MIC, and (**f**) 2 × MIC, respectively.

The permeability of the intracellular membrane of *M. luteus* was investigated using two fluorescent probes, SYTO 9 and propidium iodide (PI) from the LIVE/DEAD®BacLight™ kit, to penetrate cells. SYTO 9 freely crossed the cell membrane of all cells, stained for nucleic acids, and emitted green fluorescence. PI cannot cross the intact cell membrane, but can only cross the damaged cell membrane and stain for nucleic acid, emitting red fluorescence^37^. After *M. luteus* was stained with the fluorescent dye, the results of the fluorescence color change observed by CLSM were achieved (Fig. 2a–c). The intact cells of the control group emitted bright green fluorescence (Fig. 2a), representing cell survival. By treating *M. luteus* with MIC and 2 × MIC of PCA, the green fluorescence in the field of view was significantly reduced, and the red fluorescence was significantly enhanced, thus representing cell death. A similar mode of action was reported by Zhao et al.^38^. Plantaricin was able to inhibit some bacterial activity against *S. aureus*. As the concentration of plantaricin increased, the PI uptake significantly increased, and more cells changed from green to red. This indicates that plantaricin disrupts the cell membrane integrity of *S. aureus*. CLSM observations showed that PCA increased the permeability of the cell membrane, disrupted the integrity of the *M. luteus* cell membrane, and induced the exudation of its contents, leading to cell death^39^.

### FEG-SEM observation

The cell membrane integrity and morphological changes of PCA-treated *M. luteus* were further observed by FEG-SEM. The results are shown in Fig. 2d–f. Untreated *M. luteus* cells had an intact cell membrane, and the cells were full and smooth, showing a rounded tetragonal spherical shape (Fig. 2d). The PCA-treated cells with MIC were slightly deformed, and a small amount of mucus-like material appeared (Fig. 2e). The surface of the cells treated with PCA at 2 × MIC was wrinkled, losing the original tetragonal spherical morphology and becoming a blurry outline (Fig. 2f). These results showed that PCA somewhat reduced the integrity of the *M. luteus* cell membrane. Similar effects were examined in *Y. enterocolitica*. The degree of damage in *Y. enterocolitica* cells increased as the PCA concentration increased, as observed by scanning electron microscopy. The surface of the PCA-treated *Y. enterocolitica* by MIC was uneven and wrinkled compared with the untreated cells. After treatment with 2 × MIC of PCA, *Y. enterocolitica* lost its intrinsic rod-like bacillus morphology and appeared deformed^20^. Morphological changes in *Y. enterocolitica* and *M. luteus* cells indicated the effects of PCA on membrane integrity and revealed that PCA damaged the structure of microbial cell membranes, leading to leakage of intracellular contents and changes in cell morphology, which eventually caused cell death.

### Simulation study of PCA on the growth inhibition of *M. luteus* in pork

**Figure 3:**
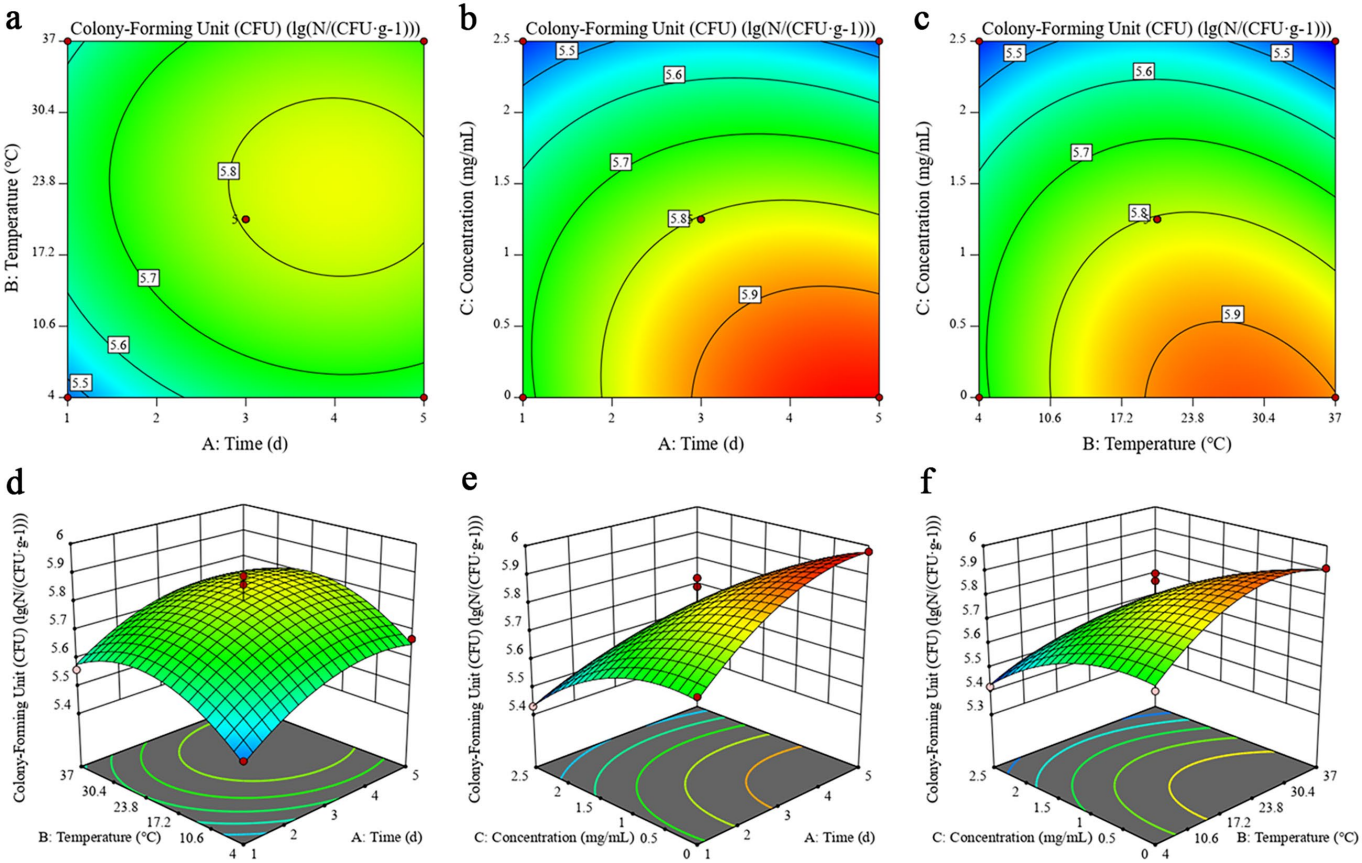
Contour plots and response surface plots illustrate the effects caused by the interaction between PCA and variables on the growth of *M. luteus* BNCC 102589 in pork. The contour plots show the effect of interactions between (**a**) time and temperature, (**b**) time and PCA concentration, and (**c**) temperature and PCA concentration on *M. luteus* growth in the pork. The response surface plots show the effect of interactions between (**d**) time and storage temperature, (**e**) time and PCA concentration, and (**f**) temperature and PCA concentration on *M. luteus* growth in pork.

*M. luteus* is prone to spoiling meat, fish, aquatic products, soybean products, and other food products. Therefore, the inhibitory effects of PCA on the growth of *M. luteus* in pork were evaluated. As shown in Table 1 and Fig. 3, the number of *M. luteus* in all groups showed an increasing trend during the 5-day storage period. However, in Fig. 3a, there was a slight decrease in CFU at 37 °C on the fifth day. This is because, during the growth process of nutrients, the bacteria consumed more and more metabolic waste, which was not conducive to bacterial growth and partial apoptosis. Therefore, there was a small decline in the total number of colonies. However, the total number of colonies treated with PCA decreased more compared to the untreated group. The number of *M. luteus* in the pork treated with PCA at the MIC decreased by 0.26–0.31 log_10_ CFU/g, and the number of *M. luteus* treated with PCA at the 2 × MIC decreased by 0.53 log_10_ CFU/g, revealing that PCA can effectively inhibit the growth of *M. luteus* in pork. A multiple quadratic regression equation was established with Design Expert 11 based on the results in Table 1 of the experimental design: *Y* = 5.8 + 0.0825 *A* + 0.0463 *B* − 0.1913 *C* − 0.0075 *AB* − 0.0675 *AC* − 0.065 *BC* − 0.0795 *A*^2^ − 0.117 *B*^2^ − 0.087 *C*^2^ *R*^2^ = 0.9615 (1), where A, B, and C represent time, temperature, and PCA concentration, respectively, and log_10_ (N/(CFU/g)) represents the logarithm of the total number of *M. luteus* colonies. Table 2 lists the MANOVA results of the established regression equations. The results suggest that the regression model was significant (*P* < 0.01). The simulated correlation coefficient *R*^2^ value was 0.9615, and the correction coefficient *R*^2^_Adj_ value was 0.9121, which suggest that the equations fit well and could more accurately predict changes in different conditions for the growth of *M. luteus* in pork. Moreover, the effects of PCA concentration, time, and temperature on the total number of colonies of *M. luteus* were significant (*P* < 0.05). There was a significant interaction between time, temperature, and PCA concentration (*P* < 0.05), as evidenced by the response surface (Fig. 3). The addition of PCA to pork in our previous study decreased the mean number of *Y. enterocolitica* by two log cycles under the low-temperature storage period^20^. In this study, PCA was found to effectively inhibit the growth of *M. luteus* in pork and its color, fat oxidation, TPA, and water migration were analyzed. This comprehensive and objective evaluation of food products with quantitative indexes provides a theoretical basis for using PCA as a natural antimicrobial agent in meat products.

**Table 2:** The regression model for predicting the best conditions for *M. luteus* inhibition with the analysis of variance (MANOVA).

Source	Sum of squares	df	Mean square	F-value	P-value
Model	0.5287	9	0.0587	19.45	0.0004
A–A	0.0545	1	0.0545	18.03	0.0038
B–B	0.0171	1	0.0171	5.67	0.0489
C–C	0.2926	1	0.2926	96.87	< 0.0001
AB	0.0002	1	0.0002	0.0745	0.7928
AC	0.0182	1	0.0182	6.03	0.0437
BC	0.0169	1	0.0169	5.59	0.0499
A^2^	0.0266	1	0.0266	8.81	0.0209
B^2^	0.0576	1	0.0576	19.08	0.0033
C^2^	0.0319	1	0.0319	10.55	0.0141
Residual	0.0211	7	0.003		
Lack of fit	0.002	3	0.0007	0.1412	0.9302
Pure error	0.0191	4	0.0048		
Cor total	0.5499	16			

### Effects of PCA on pork color

**Figure 4:**
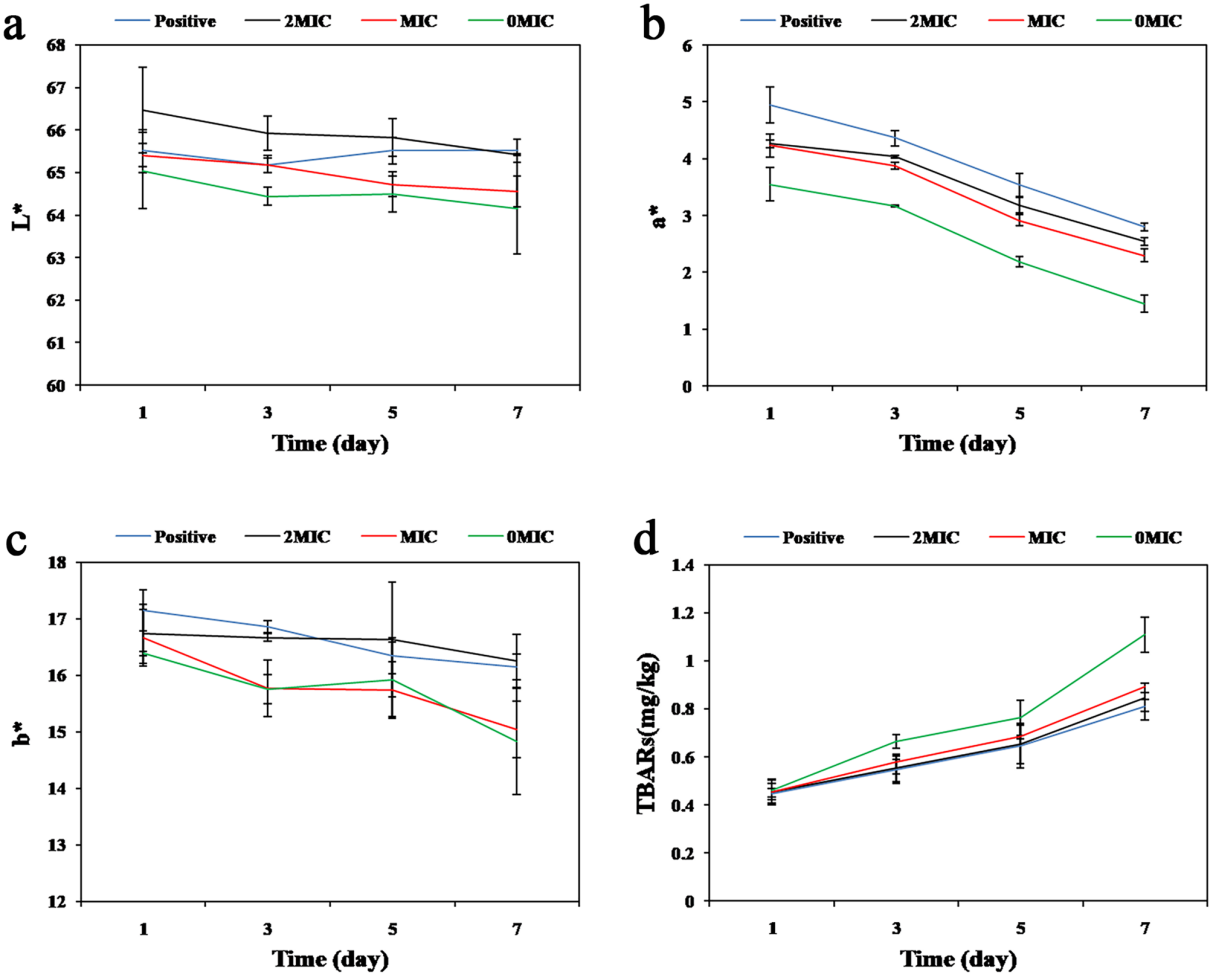
The effects of different concentrations of PCA on the pork (**a**) L* value, (**b**) a* value, (**c**) b* value, and (**d**) TBARS content. Error bars represent the standard deviation (n = 3).

The acceptability of meat depends on the variability of sensory attributes, one of which is color. In existing studies, colorimeters have been used to measure meat color^40^. Figure 4a–c show how PCA addition affects pork color at different storage periods. The trend of change of the L* (brightness value) value of the pork was relatively flat, and a* values (redness value) and b* (yellowness value) tended to decrease, while the L*, a*, and b* values of the pork in the experimental group were higher than those of the blank control group on the 1st, 3rd, 5th, and 7th days. The a* represents the freshness of the pork samples. During the late storage period, the a* value of the pork in the blank control group decreased significantly (Fig. 4b), displaying a dark red color, lack of luster, and foul odor. However, the a* values of the MIC and 2 × MIC groups were always higher than those of the 0 × MIC group. The decrease of pork a* value mainly comes from the oxidation of fats and oils, due to oxidized myoglobin becoming brown metmyoglobin^26^. In contrast, the addition of the natural antioxidant PCA showed suitable antioxidant activity, which could delay the decrease in the redness of pork and effectively maintain its freshness. Similar results were reported by Ruan et al.^41^ and Fan et al.^26^. In addition, the b* values of the low concentration group decreased sharply at 3–5 days, while the b* values of the high concentration PCA treatment were more stable (Fig. 4c). This result suggests that PCA is an effective potential natural preservative, and can improve the color of pork and increase consumer suitability.

### Effects of PCA on TBARS in pork

Pork contains considerable fat, and fat oxidation can cause the spoilage and deterioration of pork products. Lipid oxidation occurs through a free radical chain mechanism to produce end products, the main one of which is aldehydes. Additionally, the content of thiobarbituric acid reactive substances (TBARS) is indicated by the malondialdehyde content, via a reaction of malondialdehyde with thiobarbituric acid (TBA) that produces a pink dimeric compound. The above reaction is considered a vital indicator of the quality of pork products^42^. Figure 4d illustrates the effect of adding different PCA concentrations to the TBARS values of ground pork at a storage temperature of 4 °C. The initial TBARS values of all samples were approximately 0.45 mg MDA/kg. Moreover, 0 × MIC increased to 1.11 mg MDA/kg, MIC increased to 0.89 mg MDA/kg, and 2 × MIC increased to 0.85 mg MDA/kg by 7d. The TBARS value increased as the storage days of ground pork increased. Accordingly, adding PCA to pork effectively reduced the TBARS values and delayed lipid oxidation, suggesting that changes in TBARS in pork were associated with antioxidant activity and the antimicrobial effects of PCA. Huang et al.^43^ added clove and rosemary extracts, which had a significant delaying effect on lipid oxidation in pork-filled dumplings during frozen storage. The increase in the TBARS value could be due to partial dehydration and an increase in the oxidation of unsaturated fatty acids^44^.

### Effects of PCA on TPA in pork

A texture profile analysis (TPA) was conducted to graphically represent the human sensation of chewing food and evaluate changes in the textural properties of the pork based on the compression parameters^27,45^. Table 3 lists the changes in TPA parameters of ground pork during cold storage, in which hardness, adhesiveness, gumminess, and chewiness indices of the ground pork decreased as storage time increase. This result could be due to the disruption of the myogenic fiber structure caused by myogenic fiber breakage and protein hydrolysis^46^. To be specific, the hardness of 0 × MIC significantly decreased from 140.92 ± 17.23 to 73.36 ± 5.59 (*P* < 0.05), while the test group showed more stable changes, revealing that PCA could maintain changes in the hardness of ground pork during the storage period. Furthermore, the adhesiveness, gumminess, and chewiness of 0 × MIC significantly decreased at 5–7 days in the storage process (*P* < 0.05) and was lower than that of the test group. This reveals that PCA helped maintain the adhesiveness, gumminess, and chewiness of pork during the storage period. The cohesiveness and resilience in the MIC and 2 × MIC groups did not significantly change throughout the storage process. However, the three indices of springiness, cohesiveness, and resilience in the 0 × MIC group significantly increased during the late storage process (*P* < 0.05), and the meat was soft and sticky, emitting a foul odor. Thus, the effects of PCA on pork texture under 4 °C refrigeration, in descending order, were 0 × MIC > MIC > 2 × MIC.

**Table 3:** Pork texture test results with PCA added at different storage times.

TPA	Time/days	Positive control	2 × MIC	MIC	0 × MIC
Hardness	1	123.39 ± 7.95	124.07 ± 17.43	125.09 ± 25.1	140.92 ± 17.23^a^
	3	118.28 ± 16.92	122.38 ± 14.69	121.32 ± 22.98	115.18 ± 18.5^a^
	5	101.55 ± 7.97	99.76 ± 15.01	96.47 ± 10.82	105.17 ± 12.16^ab^
	7	99.82 ± 2.96	97.85 ± 7.19	93.84 ± 9.44	73.36 ± 5.59^b^
Adhesiveness	1	39.36 ± 1.23	45.72 ± 4.84^a^	45.72 ± 3.66^a^	50.75 ± 1.87^a^
	3	38.16 ± 8.39	43.00 ± 4.55^a^	42.73 ± 7.26^ab^	41.94 ± 2.92^a^
	5	34.08 ± 4.2	32.03 ± 3.28^b^	31.4 ± 7.35^ab^	38.51 ± 6.37^a^
	7	29.39 ± 1.97	28.25 ± 2.03^b^	27.86 ± 3.42^b^	21.22 ± 5.35^b^
Springiness	1	0.9 ± 0.01	0.88 ± 0.02^bc^	0.85 ± 0.02^b^	0.84 ± 0.02^c^
	3	0.91 ± 0.02	0.87 ± 0.01^c^	0.85 ± 0.01^b^	0.86 ± 0.01^bc^
	5	0.92 ± 0.01	0.92 ± 0.01^ab^	0.88 ± 0.01^ab^	0.89 ± 0.01^ab^
	7	0.92 ± 0.01	0.93 ± 0.01^a^	0.9 ± 0.02^a^	0.93 ± 0.03^a^
Cohesiveness	1	0.78 ± 0.01^b^	0.78 ± 0.04	0.75 ± 0.04	0.73 ± 0.01^c^
	3	0.79 ± 0.01^ab^	0.78 ± 0.06	0.77 ± 0.07	0.78 ± 0.03^b^
	5	0.84 ± 0.03^a^	0.83 ± 0.04	0.82 ± 0.03	0.8 ± 0.01^b^
	7	0.85 ± 0.03^a^	0.84 ± 0.05	0.82 ± 0.01	0.89 ± 0.01^a^
Gumminess	1	96.08 ± 5.26	96.71 ± 8.96	93.89 ± 13.41	102.3 ± 10.65^a^
	3	93.14 ± 11.95	95.65 ± 4.65	92.04 ± 9.33	89.39 ± 11.1^ab^
	5	85.04 ± 3.54	82.83 ± 8.08	78.99 ± 5.78	83.96 ± 9.13^ab^
	7	84.46 ± 0.37	81.85 ± 0.66	77.05 ± 7.55	65.52 ± 5.1^b^
Chewiness	1	86.92 ± 4.73	85.57 ± 10.31	79.5 ± 9.71	85.33 ± 7.02^a^
	3	84.88 ± 8.95	82.91 ± 2.85	78.62 ± 7	76.71 ± 9.81^ab^
	5	78.44 ± 4.03	76.03 ± 7.13	69.33 ± 5.11	74.65 ± 8.23^ab^
	7	77.33 ± 0.31	75.91 ± 0.76	69.61 ± 5.25	60.97 ± 6.74^b^
Resilience	1	0.38 ± 0.01^c^	0.37 ± 0.01^b^	0.37 ± 0.03^b^	0.34 ± 0.01^c^
	3	0.42 ± 0.06^bc^	0.42 ± 0.06^ab^	0.4 ± 0.04^ab^	0.42 ± 0.02^b^
	5	0.5 ± 0.04^ab^	0.5 ± 0.02^a^	0.49 ± 0.03^a^	0.45 ± 0.02^b^
	7	0.51 ± 0.02^a^	0.53 ± 0.06^a^	0.47 ± 0.01^a^	0.59 ± 0.01^a^

Each value is expressed as mean ± standard deviation (n = 3).

^a–c^Values with different letters in the same column are significantly different (P < 0.05).

The positive effect of the natural antioxidant PCA on the hardness, adhesiveness, gumminess, and chewiness of pork could be due to its antimicrobial mechanism and antioxidant activity. PCA can block the oxidation process, protecting the integrity of muscle fibers and minimizing textural changes^47^. Similar results were reported in pork treated with epicatechin gallate with chitosan^48^. Likewise, Sun et al.^49^ observed that the fennel essential oil/cinnamaldehyde Nanoemulsion could reduce the hardness of pork patties and maintain their elasticity and chewiness during storage. These results indicate that PCA has a positive effect on these textural parameters and can improve the quality of pork, which is promising since there is a demand for pork with excellent textural characteristics.

### The effects of PCA on moisture distribution in pork

**Figure 5:**
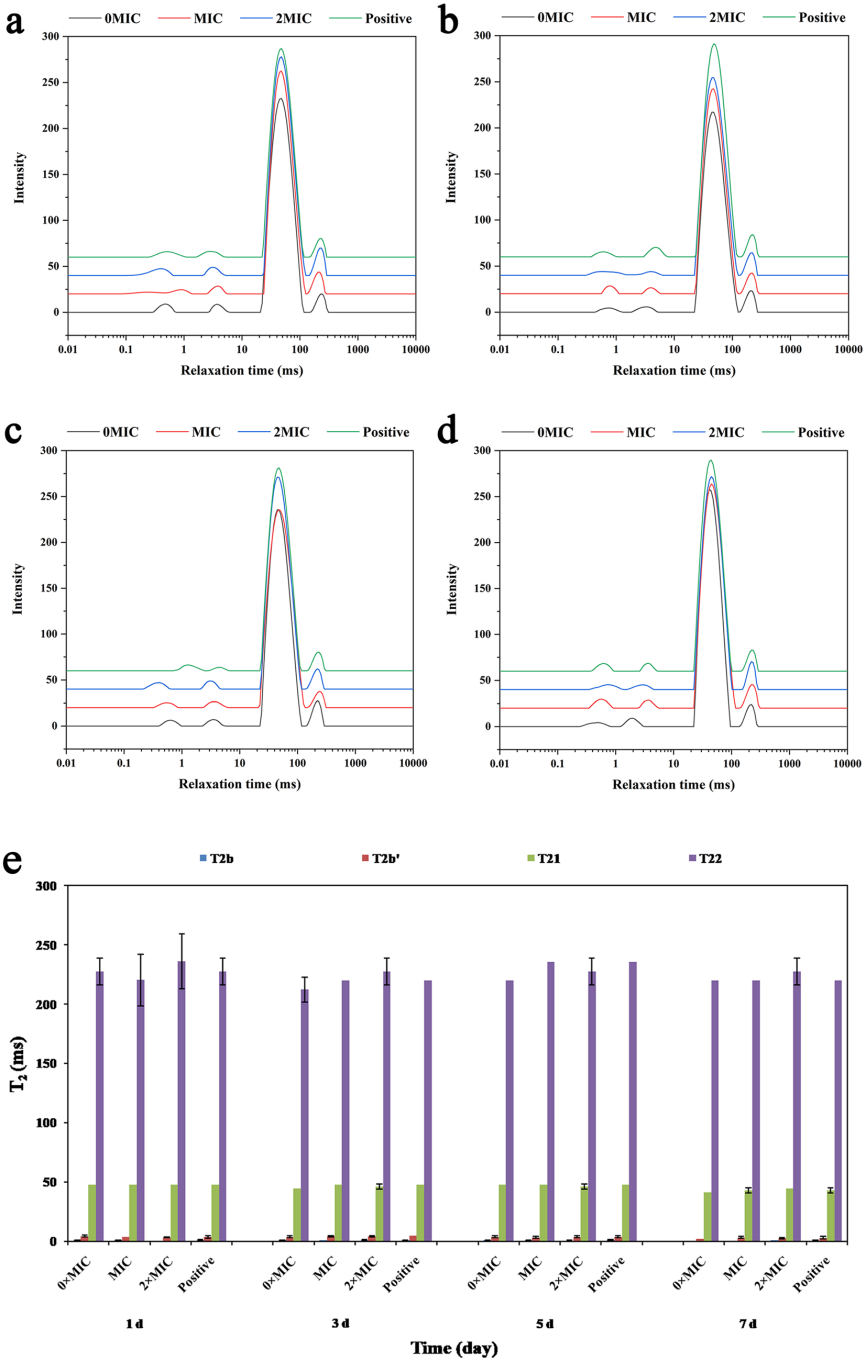
LF-NMR *T*_2_ relaxation time distribution curves for pork supplemented with PCA during different storage periods. (**a**) 1st, (**b**) 3rd, (**c**) 5th, and (**d**) 7th day. (**e**) The effect of PCA on pork water relaxation time (*T*_2_) during different storage periods. Error bars represent the standard deviation (n = 3).

Water molecules in meat and meat products primarily exist in three forms: free water, immobilized water, and bound water. For different forms of water molecules, the transverse relaxation time (*T*_2_ value) using low-field nuclear magnetic resonance (LF-NMR) technology indicates the liquidity of water molecules in meat and meat products. During relaxation time, meat can be classified into three states of water. The first is bound water, with a relaxation time (*T*_2b_) ranging from 0.1 to 10 ms. The second is immobilized water, with a relaxation time (*T*_21_) of nearly 50 ms. The third is free water, with a relaxation time (*T*_22_) of 100–1000 ms^50^. The relaxation time (*T*_2_) represents the mobility of water, and the peak area (*A*_2_) represents the moisture content. Accordingly, qualitative judgment on the existing form of water in pork can be made based on the *T*_2_ spectrum^51^. Figure 5a–d display the distribution of water in the LF-NMR transverse relaxation time (*T*_2_) of the pork with PCA added in different storage periods. The four peaks in the figure represent tight bound water (*T*_2b_), bound water (*T*_2b'_), immobilized water (*T*_21_), and free water (*T*_22_), from left to right^52^. As depicted in Fig. 5, the content of immobilized water in the pork was the highest, while the bound water and free water accounted for smaller proportions. Figure 5e displays changes in the relaxation time for the four states of moisture in pork with PCA added during the different storage periods. The figure suggests that *T*_2b_, *T*_2b'_, *T*_21_, and *T*_22_ tended to decrease during storage, while the 0 × MIC group decreased more significantly than the other groups. The fluidity of tightly bound water, bound water, immobilized water, and free water decreased as the storage time increased, while 0 × MIC achieved the greatest water fluidity. These results were likely achieved due to the irreversible damage to muscle fibers and possible damage to structures (e.g., tissues and cells), thus leading to the migration of less mobile water and the loss of free water. This shows that adding PCA was more conducive to reducing the mobility of water in pork and maintaining the quality of the product.

### Effects of PCA on sensory properties in pork

**Figure 6:**
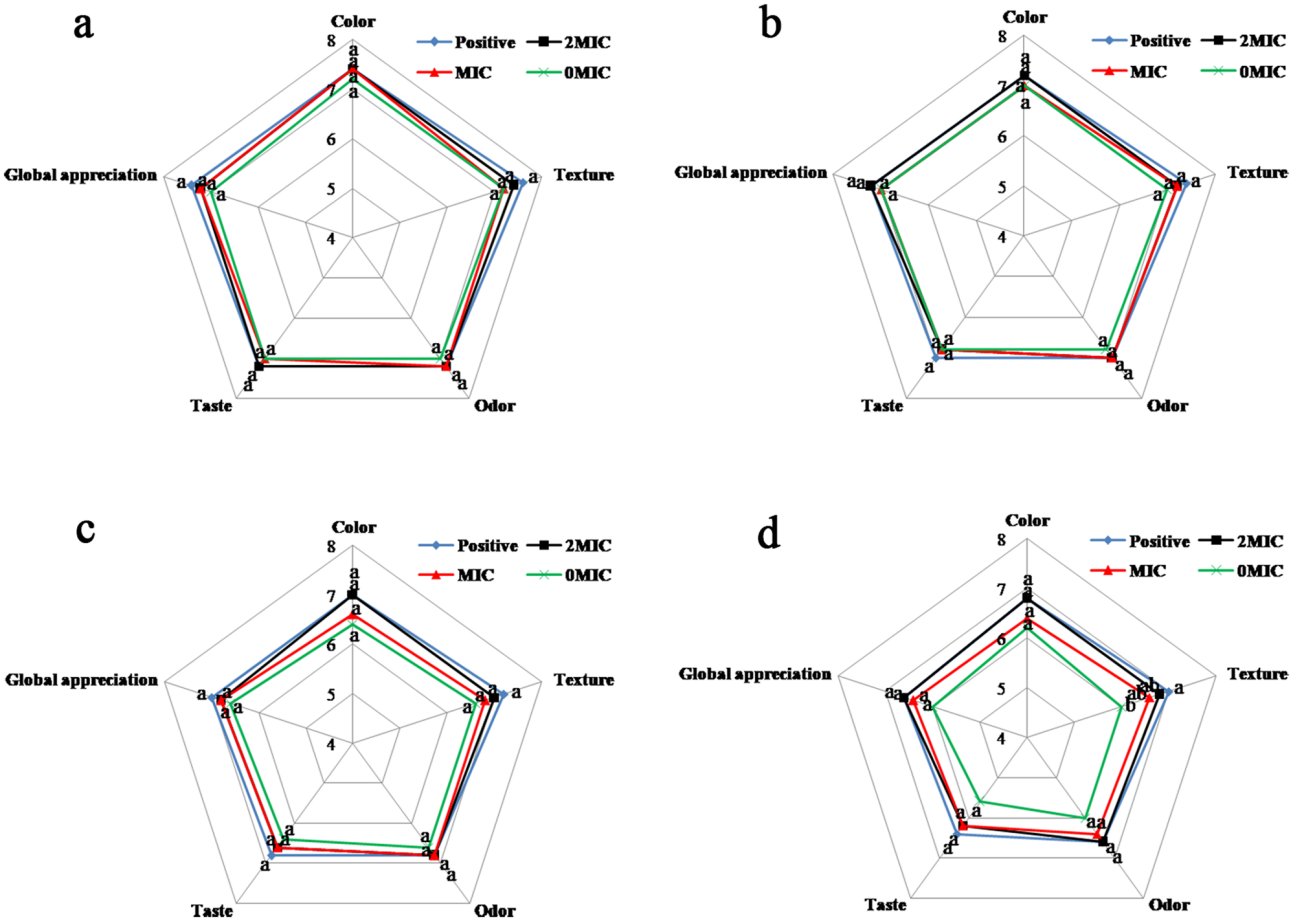
Radar plot of sensory properties of PCA applied to pork at different storage periods. (**a**) 1st, (**b**) 3rd, (**c**) 5th, and (**d**) 7th day.

The results of the sensory evaluation of pork patties are displayed in Fig. 6. As seen in the figure, the color, texture, odor, taste, and global appreciation of the control and PCA-treated pork patties were not significantly different from each other (*P* > 0.05). This indicates that the sensory characteristics of pork patties treated with PCA at concentrations of MIC and 2 × MIC were not significantly affected (*P* > 0.05). On the 7th day, the texture and taste scores of the experimental group exceeded those of the control group, indicating that the texture and taste of the pork patties in the untreated group changed, while the PCA-treated patties maintained their original texture and taste. According to the sensory evaluation results, the pork patties treated with PCA were preferable to the untreated samples. Additionally, PCA treatment did not affect the odor and taste of pork patties. Therefore, their sensory attributes are likely to be acceptable to consumers.

## Conclusions

This study suggested that PCA exhibited high antibacterial activity against *M. luteus*. PCA destroyed the integrity of cell membranes, manifested by the hyperpolarization of cell membranes, a decrease in intracellular ATP concentration, and a decrease in pH_in_. CLSM and FEG-SEM observations confirmed damage to the cell membranes. Moreover, PCA treatment significantly inhibited the increase of *M. luteus* and the growth rate of the TBARS value in raw pork during storage, improved the color and texture of the raw pork, reduced water mobility, and effectively prolonged its shelf life. Based on sensory analysis, using PCA in pork could be acceptable to consumers. Thus, PCA can serve as a natural antimicrobial agent for the food industry.

## Data Availability

Data will be available upon request. To request data from this study, please contact Sichen Liao (email address: 756743401@qq.com).
